# Analysis of changes in the association of income and the utilization of curative health services in Mexico between 2000 and 2006

**DOI:** 10.1186/1471-2458-11-771

**Published:** 2011-10-07

**Authors:** Laura G Danese-dlSantos, Sandra G Sosa-Rubí, Atanacio Valencia-Mendoza

**Affiliations:** 1 Center for Evaluation Research and Surveys, Division of Health Economics, National Institute of Public Health (INSP), Av. Universidad 655, Cuernavaca 62508, Morelos, Mexico

## Abstract

**Background:**

A common characteristic of health systems in most developing countries is unequal access to health services. As a result, members of the poorest population groups often do not receive formal attention for health services, because they cannot afford it. In 2001 in Mexico, to address income-related differences in the use of health services, the government launched a major healthcare reform, which includes a health insurance program called *Seguro Popular*, aimed at improving healthcare access among poor, uninsured residents. This paper analyzes the before and after changes in the demand for curative ambulatory health services focusing on the association of income-related characteristics and the utilization of formal healthcare providers vs. no healthcare service utilization.

**Methods:**

By using two nationally representative health surveys (ENSA-2000 and ENSANUT-2006), we modeled an individual's decision when experiencing an illness to use services provided by the (1) Ministry of Health (MoH), (2) social security, (3) private entities, or (4) to not use formal services (no healthcare service utilization).

**Results:**

Poorer individuals were more likely in 2006 than in 2000 to respond to an illness by using formal healthcare providers. Trends in provider selection differed, however. The probability of using public services from the MoH increased among the poorest population, while the findings indicated an increase in utilization of private health services among members of low- and middle-income groups. No significant change was seen among formal workers -covered by social security services-, regardless of socioeconomic status.

**Conclusions:**

Overall, for 2006 the Mexican population appears less differentiated in using healthcare across economic groups than in 2000. This may be related, in part, to the implementation of *Seguro Popular*, which seems to be stimulating healthcare demand among the poorest and previously uninsured segment of the population. Still, public health authorities need to address the remaining income-related healthcare utilization differences, the differences in quality between public and private health services, and the general perception that MoH facilities offer inferior services.

## Background

Numerous analyses around the world have found that healthcare utilization correlates with income, with lower income populations less likely to have or seek out formal healthcare. The discrepancies by socioeconomic status are particularly widespread in developing countries, including Mexico [1-5]. When ill, poorer Mexicans often do not seek formal healthcare, because they cannot afford the cost of formal healthcare [6].

Income-related differences in the use of health services in Mexico are largely because of the historical lack of access to adequate financial support for healthcare among over half of the population [1]. According to the CENSUS from the National Institute of Statistics and Geography (Instituto Nacional de Estadística y Geografía "INEGI") 57% of the population did not have any health insurance coverage in 2000 [7].

In an effort to address such shortcomings and reduce inequalities in healthcare access, in 2001 the Mexican government launched a major health reform aimed at improving healthcare access among poor, uninsured residents [7]. A key strategy was sought to increase insurance coverage among the poorest Mexicans so that they could gain affordable access to qualified public health services, health supplies, and medicines [8]. By 2005 the proportion of the population that was not covered by health insurance had decreased to 49.8%, which may have been attributed to the implementation of SP [9]. Because SP focused primarily on the very poor, it is reasonable to assume that the most notable changes in the demand for health services would have occurred mainly among members of this population.

This paper analyzes the changes in the demand for health services in 2000 and 2006 that are potentially related to the health reform implemented in 2001. We investigated the shift in an individual's selection of formal healthcare providers vs. receiving no healthcare, and the modification of income-related disparities in the demand for formal healthcare. Specifically, we made the analysis on people reporting having had a health problem in the two weeks preceding a 2000 and 2006 surveys.

Ideally, income-related variables should not be factors influencing individuals' decision to seek formal healthcare when experiencing an illness [10]. The current situation is far from ideal in Mexico, where lower income individuals have been closely associated with less utilization of formal health services [5]. Our first hypothesis is that the 2001 Mexican health reform catalyzed an uptake of health services provided by the Ministry of Health (MoH) among the poor. As a second hypothesis, we expect healthcare demand for formal health services vs. receiving no healthcare to be less differentiated for income-related conditions in 2000 and 2006.

To explore these hypotheses, we focus on two cross-sectional nationally representative health surveys of 2000 and 2006 to analyze changes in the use of curative ambulatory health services in Mexico. We refer to curative ambulatory services (not including hospitalization) because we analyze utilization based on "healthcare need" (people reporting having had a health problem in the two weeks preceding the respective 2000 or 2006 survey). We focus on curative ambulatory services (not including hospitalization) (i.e. outpatient health services provided to those who visit a healthcare facility and depart on the same day of treatment). We decided to analyze this type of health service because of their direct link with "healthcare need" as opposed to preventive healthcare services.

The next section describes the health reform, launched in 2001, that motivates this study. The methods section describes the data and the selected variables used to model individuals' demand for health services. In the results section, we interpret the associations for each of the variables considered in the model. Finally, we formulate a conclusion for each of the two hypotheses previously stated, as well as discuss additional results in the association of an income-related variable with the selection of each type of Mexican healthcare provider.

### Healthcare services in Mexico and the 2001 health reform

The structure of the Mexican health system is mainly responsible for the income-related differences in the use of health services [1]. The Mexican health system originated as a segmented model with multiple providers. Health provision was dominated by three major entities. Social security institutions represent the principal source of coverage for workers in the formal sector (workers from the formal sector covered by social insurance). This category includes the Mexican Social Insurance Institute (Instituto Mexicano del Seguro Social, "IMSS"), the Government Workers' Social Security and Services Institute (Instituto de Seguridad y Servicios Sociales de los Trabajadores del Estado, "ISSSTE"), and insurance programs for employees of state-run enterprises, such as PEMEX (petroleum), SEDENA (homeland defense), and SEMAR (marines). All of these entities are also providers of health services. Those covered by social insurance can also use private health services; however, payment is out-of-pocket. For workers outside of this category and populations in the informal sector, there are government-sponsored facilities run by the MoH (*Secretaría de Salud*). These health services are targeted to uninsured individuals but also can be used by patients with social insurance or those from more affluent classes. Government-sponsored facilities can be provided without charge or demand fees based on the socioeconomic status of the user. A third option for healthcare is the selection of private hospitals or physicians' offices with heterogeneous quality and prices, because they are used by both poor and rich groups of the population [11,12]. While a modern network of private health services for the middle and upper classes are offered to those individuals who have private insurance coverage or who can pay out-of-pocket, there are also cheaper, private health services of varying quality, such as small medical offices, health clinics, and hospitals, as well as informal providers, such as midwives and traditional healers, available to poor urban and rural groups of the population [6,13,14].

This disproportionate situation, unfavourable for the poor and informal sectors of the population, motivated a major structural reform for improving equality in healthcare access in 2001 [15]. New financial rules and incentives were introduced through the System of Social Protection in Health (SSPH). The most important component being *Seguro Popular *(SP), which was designed to support only uninsured poor families not covered by any social insurance scheme [16]. SP guarantees access to a wide package of healthcare services provided by the federal government's MoH facilities, free of charge at the time of delivery and including all medicines prescribed by licensed physicians [8].

A policy that increases health insurance coverage, such as SP, in principle may reduce the income-related restrictions to seek formal healthcare [1]. We therefore anticipate measurable indications of more equitable healthcare demand across socioeconomic groups while accounting for the health reform stimulating the use of formal health services among low-income groups of the population. As a consequence, we expect income-related factors in general to be less important in explaining formal healthcare utilization.

## Methods

We modeled an individual's decision when experiencing an illness episode by considering four options. These options included the three main types of formal health providers in Mexico - MoH services, social security services, and private entities - and a fourth reference category for not using formal services that we refer to as "no healthcare". We compared the parameters of the two models in 2000 and 2006 to explore changes in the association among individual, household, and community variables with the probability of using each of the aforementioned formal healthcare providers vs. no healthcare.

### Data sources and study sample

We used two cross-sectional, nationally representative, freely available, health surveys: *Encuesta Nacional de Salud *2000 (National Health Survey, ENSA-2000) and *Encuesta Nacional de Salud y Nutrición *2006 (National Health and Nutrition Survey, ENSANUT-2006) [17,18]. Both surveys are rich in information on household characteristics and healthcare utilization. The ENSA-2000 contains data on 190,214 individuals living in 45,870 households and was conducted between September 1999 and March 2000. The ENSANUT-2006 contains data on 206,700 individuals from 47,152 households and was conducted between November 2005 and May 2006.

The sample was restricted to those individuals who reported having had a health problem within the two weeks preceding the corresponding survey. In essence, this restricted our analysis of utilization to only include those with a "healthcare need". Of all the individuals surveyed in each study, 14% (27,177) in the 2000 survey and 11% (22,658) in the 2006 survey met the restricted criterion of having had a health problem within two weeks prior to being contacted.

We also analyzed missing values for each variable of interest in both ENSA-2000 and ENSANUT-2006 sample databases. The number of missing values for each variable of interest represents less than 3% of the sample in each survey. The only variable that had a relatively large number of missing values was level of education. This variable had 10% and 11% of missing values for 2000 and 2006 samples, respectively. Moreover, we found that observations with missing values for education level were significantly different from those that report their education: individuals who did not report their level of education either were users of MoH services or did not receive healthcare compared to those individuals who reported their education. To avoid possible bias in the results, we assumed an association between the level of education and the selection of the type of healthcare provider and substituted the missing values of education with the mean of education based on healthcare provider type. We ended up with a sample of 25,189 and 22,499 individuals for 2000 and 2006, respectively.

### Dependent variable

Those individuals who reported having had a health problem within the previous two weeks were asked whether they sought professional healthcare and, if so, what type. Those formally treated by a healthcare professional were then classified according to the type of institution they attended: the MoH, social security (SS), or private sector. Hence, utilization of health care, our dependent variable, was analyzed as a categorical variable with four possible outcomes: (1) the MoH services, (2) social security services, (3) private services and the reference category, (4) no healthcare or self-care that includes informal care.

The reference category "no healthcare" included those individuals who did not seek formal healthcare providers. Within this category, we found that the highest proportion (78%) of individuals reported receiving no care at all, with the remainder fairly evenly distributed among seeking help from (1) family, friends, or neighbors, (2) a pharmacy, (3) healers, (4) midwives, (5) herbalist, (6) homeopathy, (7) naturopathy, and (8) acupuncture.

The MoH service category included those without any insurance coverage who attend federally run health services. The MoH facilities also provide healthcare services to those families covered by the recently introduced *SP*. The SS category included those who attended institutions that cover employees of the formal sector of the economy (IMSS, ISSSTE PEMEX, SEDENA, and SEMAR).

The distribution of the analytical sample among the four different healthcare options is reported in Table 1. The health provider category with the highest frequency is no healthcare in both 2000 and 2006 surveys (38.9% and 40.3%, respectively).

**Table 1:** Descriptive statistics of the analytical samples for 2000 and 2006

		ENSA-	ENSANUT-
		2000 (n = 27,177)*	2006 (n = 22,658)*
**Dependent variable**			
**Type of health-care provider**	*Ministry of Health*	23%	18%
	*social security*	19%	17%
	*private services*	19%	25%
	*no-health-care*	39%	40%

**Individual variables**			
**Sex**	*male*	42%	46%
	*female*	58%	54%

**Age^(1)^**		26.97 [0.18]	32 [.21]
	*1 to 4 years*	19%	14%
	*5 to 20 years*	28%	26%
	*21 to 59 years*	43%	46%
	*> 59 years*	10%	15%

**Employment^(2)^**	*not employed*	74%	70%
	*employed*	26%	30%

**Social Security insurance^(3)^**	*no cover*	57%	64%
	*cover*	43%	36%

**Private insurance**	*no cover*	99%	99%
	*cover*	1%	1%

**Popular insurance^(4)^**	*no cover*	100%	88%
	*cover*		12%

**Type of health problem^(5)^**	*acute*	64%	66%
	*chronic*	6%	14%
	*injury*	4%	4%
	*other*	26%	16%

**Severity of health problem^(6)^**	*one*	3%	2%
	*two*	49%	45%
	*three*	31%	33%
	*four*	16%	17%
	*five*	2%	2%

**Household variables**			
**Indigenous language^(7)^**	*no*	93%	93%
	*yes*	7%	7%

**Head of family sex**	*male*	78%	80%
	*female*	22%	20%

**Family size**		4.73 [.02]	4.82 [.02]

**Years of education^(8)^**		7.12 [0.04]	7.69 [0.04]

**Household per person expenditure^(9)^**		4067.904 [27.27]	4272.882 [34.22]

**Community variables**			
**Type of locality^(10)^**	*rural*	23%	26%
	*urban*	26%	29%
	*metropolitan*	51%	46%

**Marginalization^(11)^**	*high*	25%	14%
	*medium*	16%	9%
	*low*	59%	77%

The statistics were adjusted for the primary unit of analysis corresponding to each survey.

[]: Std. Err.

*People reporting having had a health problem in the two weeks preceding the respective 2000 or 2006 survey

(1) The continuous and categorical statistics are presented, since the variable was modelled with a spline method.

(2) Not employed includes: unemployed, retired, students, keephouses, incapacitated and individuals who work in own informal businesses.

(3) Employment-based health insurance (IMSS, ISSSTE etc)

(4) Popular insurance was introduced until 2003

(5) Categorical variable

Acute includes: respiratory infections, Cough, Cold and Sore throat, Rheumatic fever, Ears infection, Conjunctivitis, Diarrhea, Urinary tract infections Intestinal parasitism, Headache, fever without other manifestation, Epidemic diseases (chickenpox, mea

Chronic includes: Chronic obstructive pulmonary disease (chronic bronchitis or emphysema), Cancer and malignant tumors, Tuberculosis, Heart diseases, Asthma, Renal diseases, Gastritis and Gastric ulcer, Colitis, Obesity, HIV and AIDS, Arterial hypertensio

Injury includes: Physical injuries caused by an accident and Physical injuries caused by aggression.

Other: problems reported that were not specified in the questionnaire.

(6) Self-reported severity, in the scale five is the most severe.

(7) Where at least one of the member of the family talk indigenous language.

(8) The year number one is taken when finished the first year of primary school.

(9) At 2006 prices, adjusted with the National Consumer Price Index (CPI)

(10) Type of localities: Rural.- localities with less than 2500 habitants, urban.- between 2500 to 99999 habitants, metropolitan.- more than 100,000 habitants or capital cities of each state. (ENSANUT 2006).

(11) Municipal poverty level, composite measure that includes education levels, housing conditions, income and rurality, and the factors related to living in small villages (CONAPO *Indice de Marginación *2000 and 2006)

### Independent variables

There are several key determinants for seeking healthcare [19-23]. For the analysis, we included variables at individual, household, and community levels that have been previously linked theoretically and empirically to healthcare utilization [1,24,25]. Variables at the individual level included demographic characteristics (sex, age, and employment); morbidity indicators (type of healthcare problem and perceived severity of the health problem); and healthcare access indicators (social security insurance and *SP*). Household-level variables included an income-related variable (household expenditure); social position characteristics (indigenous heritage and head of household's education); and household-specific structural factors (size of the household and sex of the head of the household). We also controlled by community-structural characteristics, such as size of the locality and marginalization level. The data was taken from ENSA-2000 and ENSANUT-2006 and then supplemented with data from the 2000 and 2006 versions of the National Survey of Household Income and Expenditures (ENIGH-2000 and ENIGH-2006).

The data for the income-related variable was generated from a sub-analysis using household expenditure predictions as a proxy. It used expenditure rather than self-reported income, because the former is a more accurate measure of constant income [26]. Throughout this paper, this variable is referred to as "household expenditure" or "expenditure level". Given the fact that household expenditure was not captured in ENSA-2000 and poorly captured in ENSANUT-2006, this variable was imputed using data from ENIGH-2000 and ENIGH-2006. The imputation was carried out using housing characteristics and household assets following methods by Moshiro et al. [27]. For both ENIGH-2000 and ENIGH-2006, the logarithm of the quarterly expenditure was regressed on household characteristics and household assets.^1 ^The estimated 2000 and 2006 regression equations were used to make a prediction of the per-trimester expenditure over our sample for ENSA-2000 and ENSANUT-2006, respectively.^2^

The basic summary statistics for all discrete and continuous variables are reported in Table 1. These descriptive statistics control for the primary unit of analysis corresponding to ENSA-2000 and ENSANUT-2006. Among those individuals who experienced a health problem, 43% in 2000 were not covered by social insurance, and 36% were not in 2006. Only 1% in both 2000 and 2006 were covered by private insurance. *SP *had not yet been introduced in 2000, but 12% of individuals in 2006 reported being beneficiaries.

We analyzed the relationship of some of the independent variables with healthcare utilization to define an adequate variable specification. Previous studies have described non-linear relationships between healthcare utilization and household expenditure, age, and education [28-30]. After testing quadratic and logarithmic specifications, both education and per capita household expenditure favoured a logarithmic transformation. We used a spline function to model the relationship between age and utilization of health services [31]. We used four partitions for age (0 to 4, 5 to 20, 21 to 59, and 60+) which are consistent with previous findings about this relationship [24,28].

### Estimation strategy

The discrete and unordered form of the dependent variable - choice of health provider (MoH services, social security services, private services) or no healthcare - motivates the use of a multinomial model [31-33]. The economic framework of the analysis relies on the idea that utilization of healthcare can be viewed as an individual behavior function of different variables. Conditional on having a medical problem, the individual chooses the healthcare provider that gives him/her the higher utility, given his/her restrictions of income and time. Each medical alternative has a different expected benefit and price/cost. The benefits minus the costs provide a *latent utility *for each alternative and each individual. Hence, the observed alternative (the selected healthcare provider) is the one that has the highest expected *latent utility *for that specific individual [34,35].

To fit the latent utility (a multivariate variable) we used a multiprobit model taking advantage of the fact that it removes the Independence of Irrelevant Alternatives (IIA) assumption. Parameters (βs) are estimated by the method of maximum likelihood with STATA 10™ Statistical Analysis Software. To facilitate the presentation and interpretation of the results, the coefficients are transformed into marginal effects. Marginal effects, in standard practice, are calculated at the mean of the explanatory variables. To compare results of two years, the marginal effects of 2000 and 2006 are calculated using the means of the 2006 variables.^3^

## Results and discussion

Table 2 shows the coefficients of the multinomial probit model. Coefficients inform the direction of the association between independent variables and the probability of using a given healthcare provider vis-à-vis using no healthcare. For the majority of the independent variables, the direction of the associations remains the same between 2000 and 2006. Women have a higher likelihood of using any kind of formal health care rather than no healthcare compared with men in both years (Table 2). This behavior has been previously reported in other studies [1,24,25,30].

**Table 2:** Multiprobit results for 2000 and 2006

**Multiprobit results**												
	
	**2000**						**2006**					
		
	**Ministry of Health**		**Social security**		**Private services**		**Ministry of Health**		**Social security**		**Private services**	
		
**Individual variables**												
**Sex**	0.1066098	***	0.010103		0.060189	*	0.098715	**	0.094658	**	0.15246	***
(Female = 1)	[3.59]		[0.31]		[1.92]		[3.06]		[2.56]		[4.94]	
	
**Age**												
*1 to 4 years*	-0.134688	***	-0.12589	***	-0.1476	***	-0.11343	***	-0.14963	***	-0.16525	***
	[-8.39]		[-6.57]		[-8.32]		[-5.68]		[-5.84]		[-8.5]	
*5 to 20 years*	-0.025155	***	-0.02164	***	-0.0262	***	-0.03907	***	-0.02916	***	-0.04613	***
	[-7.1]		[-5.32]		[-6.82]		[-9.72]		[-5.8]		[-11.78]	
*21 to 59 years*	-0.001921		0.000758		0.003567	**	0.003686	**	0.012963	***	0.00397	**
	[-1.29]		[0.47]		[2.3]		[2.26]		[7.23]		[2.58]	
*> 59 years*	-0.002066		0.001699		0.0019		-0.00065		0.003731		0.006274	*
	[-0.58]		[0.45]		[0.53]		[-0.18]		[1]		[1.85]	
	
**Social Security**	-0.245047	***	1.760822	***	-0.1487	***	-0.53418	***	2.001224	***	-0.25553	***
(with SS = 1)	[-8.14]		[51.09]		[-4.85]		[-13.72]		[48.53]		[-7.77]	
	
**Popular Security**							0.754548	***	-0.16032	**	-0.34276	***
(with SP = 1)							[20.22]		[-2.12]		[-7.62]	
	
**Employment**	-0.162625	***	-0.14039	***	-0.11082	**	-0.30882	***	-0.2661	***	-0.02543	
(employed = 1)	[-4.25]		[-3.45]		[-2.83]		[-7.4]		[-5.85]		[-0.65]	
	
**Type of problem**												
*Chronic vs. Acute*	0.9999034	***	1.025038	***	0.818915	***	0.854733	***	1.016861	***	0.686729	***
	[16.5]		[16.35]		[12.23]		[17.79]		[19.98]		[14.93]	
*Leisure vs. Acute*	0.5212124	***	0.557148	***	0.217191	**	0.617857	***	0.667024	***	0.179247	**
	[7.46]		[7.4]		[2.78]		[8.52]		[8.63]		[2.47]	
*Other vs. Acute*	0.5034256	***	0.613516	***	0.550772	***	0.385227	***	0.598235	***	0.402879	***
	[15.04]		[16.8]		[15.84]		[9.12]		[12.71]		[10.03]	
	
**Severity**												
*two vs. one*	0.2510569	**	0.380829	***	0.222244	**	0.357855	***	0.27125	**	0.253324	**
	[2.99]		[4.06]		[2.54]		[3.24]		[2.32]		[2.55]	
*three vs. one*	0.4361775	***	0.545099	***	0.431442	***	0.64665	***	0.681039	***	0.70249	***
	[5.1]		[5.72]		[4.86]		[5.81]		[5.78]		[7.01]	
*four vs. one*	0.9586456	***	1.108386	***	1.007111	***	1.09434	***	1.133015	***	1.205795	***
	[10.77]		[11.17]		[10.9]		[9.53]		[9.3]		[11.59]	
*five vs. one*	1.263498	***	1.218258	***	1.066955	***	1.438717	***	1.598196	***	1.493361	***
	[9.31]		[7.89]		[7.4]		[9.41]		[9.83]		[10.41]	
	
**Household variables**												
**Household expenditure**	-0.060062	**	0.248891	***	0.736517	***	-0.20721	***	0.042949		0.433875	***
log(expenditure)	[-2.33]		[8.47]		[26.85]		[-6.3]		[1.21]		[14.53]	
	
**Head of family sex**	0.0052943		-0.09514	**	-0.10713	**	-0.04915		-0.12671	**	-0.14456	***
(female = 1)	[0.17]		[-2.6]		[-3.13]		[-1.31]		[-2.86]		[-3.96]	
	
**Education (head of family in years)**	0.0330706	**	0.108187	***	0.209508	***	0.00561		0.063839	**	0.13855	***
	[1.59]		[4.58]		[9.33]		[0.23]		[2.17]		[5.62]	
	
**Indigenous**	0.3755547	***	-0.0241		-0.00235		0.024288		-0.1004		-0.07187	
**language **(= 1)	[8.08]		[-0.36]		[-0.04]		[0.44]		[-1.31]		[-1.22]	
	
**Family size**	0.0219594	**	0.034381	***	0.098925	***	-0.03173	***	0.004301		0.047423	***
	[2.72]		[3.66]		[11.32]		[-3.51]		[0.41]		[5.51]	
	
**Community variables**												
**Locality**												
*Urban vs. rural*	-0.237031	***	0.250429	***	0.124334	**	-0.15641	***	0.17394	**	0.171471	***
	[-6.89]		[5.61]		[3.13]		[-3.67]		[2.86]		[3.7]	
*Metropolitan vs. rural*	-0.434301	***	0.097808	*	0.040713		-0.46282	***	0.107602	*	0.154387	**
	[-9.63]		[1.86]		[.81]		[-9.76]		[1.72]		[3.13]	
	
**Marginalization**												
*Low vs. High*	-0.066694	*	0.079887	*	-0.04052		0.12739	**	0.388002	***	0.192217	***
	[-1.77]		[1.83]		[-0.96]		[2.47]		[4.75]		[3.34]	
*Medium vs. High*	-0.124184	***	-0.10117	**	0.118674	**	0.167172	**	0.245294	**	0.287282	***
	[-3.26]		[-2.05]		[2.76]		[3.03]		[2.7]		[4.59]	

***: P < 0.001, **: P < 0.05, *: P < 0.1

[]: corresponding z estimate

For analyzing and comparing the magnitude of the effects, Table 3 shows the 2000 and 2006 marginal effects derived from the multinomial probit models calculated at 2006 means. One finding consistent for both 2000 and 2006 is that those individuals covered by social security show a higher probability of demanding social security facilities compared with those who are not covered. However, this result is not as remarkable as we would expect to find it, taking into account that the probability of seeking formal health services among those individuals covered by social security services is less than 50% in any year: 36.92% in 2000 and 40.31% in 2006 (Table 3). These low probabilities may reflect factors that serve as disincentives for individuals to seek care at these facilities, such as long waiting lines and perceptions of poor quality, which have been previously documented. Health services provided through the MoH targeting the uninsured population and health services provided by one of the main providers of social insurance services for the formal workers named Instituto Mexicano del Seguro Social (IMSS) have been characterized by their high waiting times [36-39]. One alternative to the high costs that represent long waiting times in public institutions is the use of private health services as long as the utility^4 ^of private health services are higher than the utility of using public services.

**Table 3:** Multiprobit Marginal Effects for 2000 and 2006

**Multiprobit Marginal Effects**																
		
	**2000^**								**2006**							
		
	**Ministry of Health**		**Social security**		**Private services**		**No healthcare**		**Ministry of Health**		**Social security**		**Private services**		**No healthcare**	
	
**Individual variables**																
**Sex**	0.021993	***	-0.00579		0.005341		-0.02154	**	0.007413		0.003258		0.02763	***	-0.0383	***
(Female = 1)	[3.33]		[-1.22]		[0.89]		[-2.86]		[1.12]		[0.7]		[3.98]		[-4.84]	
	
**Age**																
*1 to 4 years*	-0.01756	***	-0.00808	**	-0.01778	***	0.043427	***	-0.0078	**	-0.00978	**	-0.02756	***	0.045135	***
	[-5.03]		[-2.94]		[-5.31]		[10.11]		[-1.99]		[-3.06]		[-6.53]		[8.68]	
*5 to 20 years*	-0.00346	***	-0.00126	**	-0.0031	***	0.007823	***	-0.00431	***	-0.00073		-0.00765	***	0.012682	***
	[-4.34]		[-2.11]		[-4.15]		[8.63]		[5.21]		[-1.13]		[-8.58]		[12.68]	
*21 to 59 years*	-0.00076	**	7.35E-05		0.000874	**	-0.00019		7.58E-05		0.001495	***	0.000197		-0.00177	***
	[-2.27]		[0.32]		[2.94]		[-0.5]		[0.22]		[6.55]		[0.56]		[-4.53]	
*> 59 years*	-0.00073		0.000297		0.000486		-5.6E-05		-0.00076		0.000277		0.001509	**	-0.00102	
	[-0.92]		[0.55]		[0.72]		[-0.06]		[-1.06]		[0.6]		[1.99]		[1.18]	
	
**Social Security**	-0.14608	***	0.369284	***	-0.09722	***	-0.12599	***	-0.1746	***	0.403158	***	-0.13506	***	-0.0935	***
(with SS = 1)	[-24.77]		[62.83]		[-18.47]		[-17.62]		[-29.78]		[54.51]		[-21.71]		[-11.58]	
	
**Popular Security**									0.232767	***	-0.03784	***	-0.13503	***	-0.0599	***
(with SP = 1)									[24.11]		[-4.91]		[-17.2]		[-5.8]	
	
**Employment**	-0.0262	***	-0.01054	*	-0.00755		0.044295	***	-0.05699	***	-0.02419	***	0.027226	**	0.053955	***
(employed = 1)	[-3.12]		[-1.84]		[-1.01]		[4.65]		[-7.16]		[-4.58]		[3]		[5.41]	
	
**Type of problem**																
*Chronic vs. Acute*	0.137335	***	0.085518	***	0.052153	***	-0.27501	***	0.099514	***	0.090829	***	0.050009	***	-0.24035	***
	[9.14]		[7.53]		[4.18]		[-22.55]		[8.91]		[10.3]		[4.69]		[-24.47]	
*Leisure vs. Acute*	0.090074	***	0.06075	***	-0.01717		-0.13365	***	0.108452	***	0.071653	***	-0.0404	**	-0.13971	***
	[5.06]		[4.35]		[-1.2]		[-8.1]		[6.01]		[5.2]		[-2.67]		[-8.59]	
*Other vs. Acute*	0.054003	***	0.054833	***	0.058673	***	-0.16751	***	0.031918	***	0.057372	***	0.045135	***	-0.13442	***
	[6.78]		[8.83]		[8]		[-21.35]		[3.49]		[7.69]		[4.74]		[-13.91]	
	
**Severity**																
*two vs. one*	0.029152		0.041013	**	0.015706		-0.08587	***	0.052433	**	0.014185		0.026005		-0.09262	***
	[1.49]		[2.84]		[0.9]		[-4.22]		[2.18]		[0.91]		[1.09]		[-3.8]	
*three vs. one*	0.051851	**	0.05129	***	0.040761	**	-0.1439	***	0.065949	**	0.041302	**	0.100401	***	-0.20765	***
	[2.52]		[3.25]		[2.22]		[-7.15]		[2.61]		[2.42]		[3.97]		[-8.97]	
*four vs. one*	0.10071	***	0.094324	***	0.098684	***	-0.29372	***	0.097298	***	0.061064	**	0.164989	***	-0.32335	***
	[4.34]		[4.82]		[4.57]		[-17.04]		[3.41]		[3]		[5.73]		[-17]	
*five vs. one*	0.16614	***	0.084353	**	0.063805	**	-0.3143	***	0.106793	**	0.099929	**	0.152847	***	-0.35957	***
	[4.52]		[2.76]		[2.03]		[-14.86]		[2.79]		[3.14]		[3.98]		[-21.73]	
	
**Household variables**																
																
**Household expenditure**	-0.07734	***	0.014047	***	0.153799	***	-0.09051	***	-0.08206	***	-0.00486		0.125839	***	-0.03892	***
log(expenditure)	[-13.39]		[3.27]		[29.07]		[-13.76]		[-12.19]		[-1.09]		[18.61]		[-5.02]	
	
**Head of family sex**	0.013066	*	-0.01124	**	-0.01952	**	0.017693	**	0.004096		-0.00965	*	-0.02778	***	0.03334	***
(female = 1)	[1.79]		[-2.19]		[-3.07]		[2.15]		[0.53]		[-1.8]		[-3.47]		[3.57]	
	
**Education (head of family in years)**	-0.01124	**	0.007633	**	0.038844	***	-0.03524	***	-0.01164	**	0.002842		0.032566	***	-0.02376	***
	[-2.42]		[2.2]		[8.79]		[-6.63]		[-2.28]		[0.76]		[5.81]		[-3.82]	
	
**Indigenous language (= 1)**	0.102686	***	-0.02229	**	-0.02726	**	-0.05314	***	0.01464		-0.01136		0.014471		0.012891	
	[8.53]		[-2.55]		[-2.47]		[-4.17]		[1.26]		[-1.24]		[-1.22]		[0.89]	
	
**Family size**	-0.00293		0.000613		0.018561	***	-0.01625	***	-0.01102	***	-0.00027		0.01392	***	-0.00318	
	[-1.63]		[0.45]		[10.89]		[-7.79]		[-5.97]		[-0.2]		[7.44]		[-1.44]	
	
**Community variables**																
**Locality**																
*Urban vs. rural*	-0.0772	***	0.048644	***	0.032454	***	-0.0039		-0.05342	***	0.022684	**	0.04903	***	-0.0183	
	[-10.89]		[6.63]		[4.02]		[-0.42]		[-6.67]		[2.71]		[4.45]		[-1.59]	
*Metropolitan vs. rural*	-0.11434	***	0.034634	***	0.034297	***	0.045408	***	-0.11878	***	0.024422	**	0.070309	***	0.024048	*
	[-11.51]		[4.53]		[3.55]		[3.88]		[-12.91]		[3.02]		[6.23]		[1.95]	
	
**Marginalization**																
*Low vs. High*	-0.01771	**	0.017216	**	-0.00721		0.007711		0.001694		0.038192	***	0.024392	*	-0.06428	***
	[-2.08]		[2.8]		[-0.88]		[0.79]		[0.16]		[4.2]		[1.89]		[-4.54]	
*Medium vs. High*	-0.03394	***	-0.01502	*	0.039555	***	0.009403		0.005668		0.01598		0.051781	***	-0.07343	***
	[-4.26]		[-2.21]		[4.35]		[0.92]		[0.49]		[1.23]		[3.33]		-4.89	

***: P < 0.001, **: P < 0.05, *: P < 0.1

[]: corresponding z estimate

^Marginal effects of 2000 calculated at 2006 means

The probability of no healthcare increases, and the likelihood of formal healthcare utilization decreases among employees from the formal sector (4.42% in 2000, and 5.39% in 2006) compared to the unemployed population (as the reference category of the variable employment) (see appendix of Table 1). An opposite behavior should be noted in regards to private services in 2006 in this category: an employed individual has 2.72% more probability of seeking private services than an unemployed individual (Table 3).

The marginal effects for the type of health problem are similar in 2000 and 2006. Those who experienced a chronic health problem or injuries were more likely to use formal healthcare compared to those who reported having an acute problem. This finding has been reported previously for the elderly population in Mexico [40].

We also found that those who perceived a higher severity of illness use more formal health services than those who perceived a lower severity of illness. An exception occurs for private healthcare services, since the likelihood of using private health services declines when a problem changes from severe to very severe. This is due largely to the assumption that households with very serious health problems will ultimately be unable to afford private expenditures and, thus, must utilize public services [25].

As expected, education level was found to be significantly associated with utilization of any type of formal healthcare provider. Increasing levels of education increases the probability of using formal healthcare services for all types of formal healthcare providers in both 2000 and 2006 (Table 3).

Compared with people living in rural areas, individuals from urban or metropolitan localities are significantly more likely to use social security and private services and less likely to use services from the MoH (Table 3). These results may reflect the structure of the labour market in Mexico. Urban areas have more extensive and developed formal labour markets with access to social security services and a greater variety of private health services. In contrast, the majority of the labour market in rural areas is informal, resulting in a less extensive presence of social insurance services and more participation of the MoH health services [40].

In both years (2000 and 2006), people living in highly marginalized communities were more likely to use MoH services than their less marginalized counterparts (Table 3). This may reflect the fact that communities with greater social deprivation have a lower participation in the formal labour market and a more limited access to social insurance services [40].

### Household expenditure level

The marginal effect of Table 3 is interpreted as a change in the probability of choosing a given healthcare provider associated with a percentage increase in per capita household expenditure.

**Figure 1:**
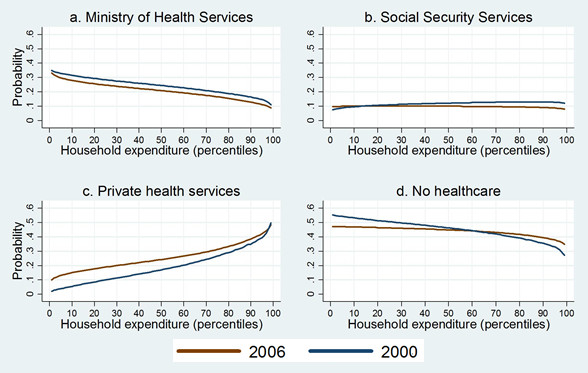
**Probability of using healthcare services by type of provider**.

The association of lower levels of household expenditure with no healthcare diminishes between 2000 and 2006. While a one-percentage increase in per capita expenditure decreases the probability of no healthcare by 9.05% in 2000, it decreases the probability of no healthcare by 3.89% in 2006 (Table 3). Figure 1 demonstrates the smoother slope of predicted probabilities in 2006 compared with 2000 in Figure 1 (see d. no healthcare). As we can see, differences in household expenditure level play a less important role in a household's decision to seek healthcare services in 2006 than in 2000.

We found that members of the worst-off households in terms of household expenditure were more likely to choose formal health services over no healthcare in 2006 than in 2000. Figure 1 (see d. no healthcare) shows that individuals in the first decile of household expenditure have a lower estimated probability of no healthcare in 2006 compared with 2000.

Even though an increase in household expenditure is associated with an increase in the probability of using formal services, the opposite trend occurs in regards to utilization of MoH services. Higher household expenditure level is associated with a reduction in the probability of using this type of provider, a finding that confirms that MoH services are perceived as an inferior good [25,41]. In both 2000 to 2006, this effect was significant and became more notable across the years (-7.73% in 2000 to -8.20% in 2006).

Household expenditure has neither a significant nor an important association with the probability of using social security services (see Table 3). The figure that corresponds to the probability of using social security services in Figure 1 shows flat curves for 2000 and 2006 in regards to predicted probability across household expenditure levels. These results reinforce assumptions that health services provided through the social security health system reduce inequality in healthcare access and do not differentiate across income groups.

For both 2000 and 2006, the probability of using private health services increases with improvements in household expenditure levels, yet for 2006, this positive association diminishes (15.38% in 2000 and 12.58% in 2006, see Table 3). Therefore, expenditure level differences are less distinct in 2006 with respect to 2000 (see Figure 1c. private services). This result may reflect a more heterogeneous private sector in 2006 with respect to 2000, including greater availability of cheaper medications and consultations that are more affordable to a greater proportion of the population [42]. The sharply increased access to relatively cheap providers of generic medicines, along with the low-cost clinics in the health market in Mexico that began in the late 1990s in the wake of legal and policy changes, could be associated with the greater demand for private health services in 2006 compared with 2000 [43].

### Robustness of results

To determine whether or not the findings using the imputation of household expenditure (as a proxy of household income) were robust with other income-related proxy measures, we ran the same model using an asset index as a proxy of household welfare. By using principal components approach, we created an index of wealth using information of the assets available in each household. Results show that both proxy measures of income level (expenditure imputation and asset index) provide consistent results for each type of healthcare provider. Changes in the probability of using one type of healthcare provider across income-related levels (from 2000 to 2006) were similar for both proxy measures (results not shown - for this information please write to the corresponding author).

### Analysis for more severe conditions

To analyze if those who are in need indeed use healthcare, we ran the same model restricting the sample for those who reported a very severe health condition (340 and 418 individuals in 2000 and 2006, respectively) to observe the association of household expenditure level with the selection of health provider.

Household expenditure was significantly associated with the use of health services in 2000; however, the association was not statistically significant in 2006. Households with lower expenditure levels had a greater likelihood of receiving no healthcare compared to those with higher expenditure levels. These differences are remarkably greater in 2000 compared to 2006. One possible explanation for this is that by 2006, household expenditure levels have become less associated with the non-use of healthcare services.

**Figure 2:**
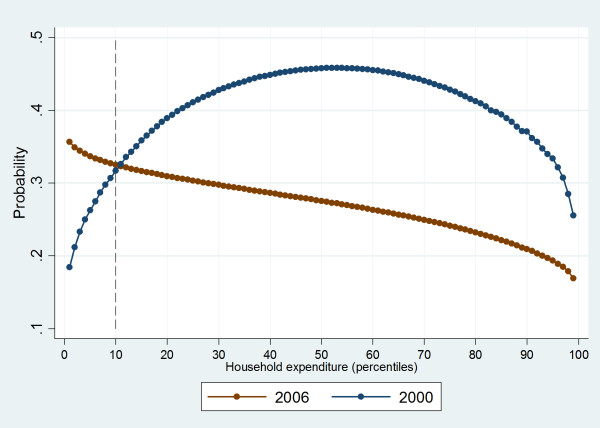
**Probability of using Ministry of Health Services (sub-sample with very severe health conditions)**.

Moreover, those who are within the first decile for household expenditure had a higher probability of using public services provided by the MoH in 2006 compared to 2000 (see Figure 2). It is clear that among those with a very severe health condition within the poorest population groups (first expenditure-decile), the probability of using federally run health services provided by the MoH have increased between the two years 2000 and 2006 (see Figure 2).

## Conclusions

A common concern in developing countries is to ensure that those individuals who are in need have access and are able to utilize qualified health services, irrespective of their socioeconomic or employment statuses. This study contributes to international empirical research regarding income-related differences in healthcare utilization behavior as a response to a healthcare need. Moreover, it helps monitor the associated effects of the 2001 healthcare reform in Mexico, a development that was specifically aimed to both reduce the barriers to access of qualified healthcare and eliminate differences in healthcare utilization across socioeconomic or employment statuses.

Our findings show that this effort has been somewhat successful. Significant gaps persist, with members of the worst-off populations still less likely to utilize formal healthcare services. However, from 2000 to 2006, there has been an increase in the probability of healthcare utilization among poorer individuals with a severe health condition. Our findings presented here suggest that *SP *is already a driver of increasing healthcare utilization provided by the MoH among the poorest groups of population. It seems that the Mexican government's efforts have contributed in overcoming disparities in healthcare utilization by income groups.

On a less positive note, an important finding is that health services provided by the MoH are still perceived as an inferior good. This is apparently even stronger and more significant for 2006 in comparison to 2000. The richer individuals are the more eager to avoid using MoH even though they must pay out of pocket. Thus, although steps have been taken to cover the poorest families, important challenges remain to be addressed, especially regarding citizens' confidence in public services.

Additionally, the findings from 2000 and 2006 show significantly increased utilization of private health services among members of low- and middle-income groups. This increased utilization is contributing to the 2006 Mexican population being less differentiated across income groups to receive no healthcare as compared to the year 2000. This trend is probably because of the expanded availability of generic medicines within the period of analysis; however, further analysis will need to be conducted to confirm this. In contrast, the use of social security services remained constant: the utilization of those services is not differentiated across income groups, which reinforces evidence that social security insurance is an effective strategy that reduces inequality. The observed changes in the use of ambulatory services demonstrate less differentiated healthcare utilization by income groups in 2006.

Still, there are challenges that the Mexican government needs to recognize and address in order to more effectively increase healthcare access and ensure equitable distribution. *SP *may be increasing healthcare demand in the poorest sector, but, as previously shown in the result section, major gaps that could be related to the quality of health services provided through the MoH must be addressed.

### Limitations

There are several limitations in this study. One is that it is impossible to gauge the impact of *SP *implementation on changes in healthcare utilization in 2000 and 2006. This article does not report results from an impact evaluation, but instead, it describes an analysis that monitors the changes in healthcare utilization for different healthcare providers present in Mexico. Although progress in efforts to ensure greater equality in healthcare access are mentioned here, it is not possible to truly identify the reasons behind the changes mentioned, which is a limitation.

Moreover, this study analyzes changes in healthcare utilization for individuals that faced a health problem in the two weeks preceding the 2000 and 2006 surveys, but it does not consider changes in healthcare outcomes. There have been some studies that have tried to measure the impact of *SP *on health in 2006. Although some of them have reported some impact on the control of blood pressure [44] and diabetes [45] among those enrolled in *SP *compared to those not enrolled, some other studies have not found any effect on health indicators [15]. Questions about the effect on outcomes need to be addressed in further analysis using more recent data.

Finally, another limitation of the study is the absence of supply-side variables for the four different types of healthcare providers. These variables were not available within ENSA and ENSANUT, nor are they available in a complementary dataset. However, waiting time and quality of healthcare are inherent characteristics for the different providers. Individuals consider waiting time as an inherent cost that varies for each type of health provider.

## Competing interests

The authors do not have any financial or non-financial competing interests and are solely responsible for the contents expressed in this paper.

## Authors' contributions

LGDS assisted with the study design, conducted the analysis of the data, interpreted the results, and led the writing of the manuscript. AVM conceived the study, conducted the study design, interpreted the results, and contributed to the writing of the manuscript. SGSR assisted with the study design, interpreted the results, critically revised the content, and also contributed to the writing of the final manuscript. All authors have read and approved the final manuscript.

## Pre-publication history

The pre-publication history for this paper can be accessed here:

http://www.biomedcentral.com/1471-2458/11/771/prepub

## Endnotes

^1^These characteristics and assets included the household's relation to its living quarters (ownership, rent, etc.); possession of items such as motor vehicles, number of rooms in the dwelling; presence of electricity, drainage, and running water; and types of ceiling and floor materials. The adjusted R^2 ^was 0.50 and 0.54 for the 2000 and 2006 regressions, respectively.

^2^This variable was compared with national distribution of income to ensure it was a reliable indicator.

^3^By using 2006 means for marginal effects of both years, we are assuming a hypothetically constant "representative 2006 individual" at both points in time. In this way, the marginal effect differences, between years 2000 and 2006, are free from changes across time of the independent variables.

^4^Each medical alternative (social insurance -IMSS, ISSSTE, etc.-, MoH, private health services and no healthcare) has a different expected benefit and price/cost. The benefits minus the costs provide a latent utility for each alternative and each individual.
